# Four new species of the subfamily Homoneurinae (Diptera, Lauxaniidae) from southwestern China

**DOI:** 10.3897/zookeys.953.53976

**Published:** 2020-07-27

**Authors:** Wenliang Li, Ling Qi, Ding Yang

**Affiliations:** 1 College of Forestry, Henan University of Science and Technology, Luoyang 471023, China Henan University of Science and Technology Luoyang China; 2 Department of Entomology, China Agricultural University, Beijing 100193, China China Agricultural University Beijing China

**Keywords:** acalyptrate flies, *
Cestrotus
*, *
Phobeticomyia
*, *
Prosopophorella
*, Oriental region, taxonomy

## Abstract

Four species of Homoneurinae from southwestern China are described as new to science: *Cestrotus abdominalis***sp. nov.**, *Cestrotus albifacies***sp. nov.**, *Phobeticomyia motuoensis***sp. nov.**, and *Prosopophorella longa***sp. nov.** An updated key to the species of the genera *Cestrotus*, *Phobeticomyia*, and *Prosopophorella* recorded in China is presented.

## Introduction

The family Lauxaniidae is a large family of the Acalyptratae. There are more than 170 genera and nearly 2100 described species, distributed worldwide except for Antarctica. The subfamily Homoneurinae of Lauxaniidae was established by Stuckenberg in 1971 (Stuckenberg 1971a) on the basis of studies including 28 genera and more than 780 species worldwide, of which seven genera and 240 species are recorded from China alone.

Among the seven genera of this subfamily in China, *Homoneura* Van der Wulp, 1891 is the largest, containing more than 200 species; Shi and Yang (2014) described 20 species. The genus *Dioides* Kertész, 1915 in China contains six species; Shi et al. (2009a) described five of them. The genus *Noonamyia* Stuckenberg, 1971 (Stuckenberg 1971b) in China contains seven species: Shi and Yang (2009a) described two species, and we described three species (Li et al. 2020). *Wawu* Evenhuis, 1989 is the smallest genus of the Homoneurinae in China, containing a single species. The three remaining genera of Homoneurinae in China are *Cestrotus* Loew, 1862, *Phobeticomyia* Kertész, 1915, and *Prosopophorella* de Meijere, 1917, containing nine, five, and three species respectively after this research; most of them are distributed in southwestern China, especially Yunnan Province, Hainan Province, and Guangxi Province (Shi et al. 2009c; Shi et al. 2009b; Shi and Yang 2009b).

Southwestern China with a rich biodiversity is located in the Oriental region and includes the Sichuan Basin, Yunnan-Guizhou Plateau, Southern Qinghai-Tibet Plateau and Western Guangxi-Guangdong Hills. It has obvious karst landforms and river valley landforms, large altitude differences and a complex terrain. It encompasses a variety of high-rainfall climates including subtropical monsoon climates, plateau mountain climates, and tropical rainforest climates. There are more than 50 national and provincial nature reserves, each with complete ecological preservation, high vegetation abundance, and rich humus soils and fungi. These are the reasons why most species of Lauxaniidae are distributed in southwestern China.

In this article, four new species are described from this diverse area of China: *Cestrotus abdominalis* sp. nov., *Cestrotus albifacies* sp. nov., *Phobeticomyia motuoensis* sp. nov., and *Prosopophorella longa* sp. nov. An updated key to the species of genus *Cestrotus*, *Phobeticomyia*, and *Prosopophorella* in China, which is based on the keys of Shi et al. (2009c), Shi et al. (2009b), and Shi and Yang (2009b) is presented.

## Materials and methods

Genitalia preparations were made by removing and macerating the apical portion of the abdomen in cold saturated NaOH for 6 h, then rinsing and neutralizing them for dissection and study. After examination in glycerin, they were transferred to fresh glycerin and stored in a microvial on a pin below the specimen or moved to an ethanol tube together with the alcohol specimens. Specimens examined were deposited in the Entomological Museum of China Agricultural University, Beijing, China (CAUC).

The general terminology follows Gaimari and Silva (2010) and Shi and Yang (2014).

## Taxonomy

### Key to species of the genera *Cestrotus*, *Phobeticomyia*, and *Prosopophorella* in China

**Table:** 

1	Face shining with distinct spherical protuberance, at least half of base dark brown	**genus *Phobeticomyia* (Kertész, 1915) 2**
–	Face with a median protuberance on ventral margin or slight convex on the middle or convex and with complex bands	**6**
2	Wing with 1-2 narrow hyaline subapical bands (constricted at middle in some species) in m_1_ cell; surstylus not as follows	**3**
–	Wing without narrow hyaline subapical stripe in m_1_ cell; surstylus with one small triangular apical process and one curved inner process with apical tooth	***Ph* . *spinosa* (Sasakawa, 1987)**
3	Wing (Fig. 22) with two narrow hyaline subapical bands in m_1_ cell, three hyaline spots between R_2+3_ and R_4+5_; male genitalia (Figs 26–30): syntergosternite semicircular; hypandrium V-shaped, with a pair of short inner process on sides; gonopod with setulae basally, curved apically	***Ph. motuoensis* sp. nov.**
–	Wing with one narrow hyaline subapical band (constricted at middle in some species) in m_1_ cell; male genitalia not as above	**4**
4	Wing with a hyaline spot between *dm*-*cu* and subapical band in m_1_ cell, and with a round median spot near CuA_1_ in cua_1_ cell	**5**
–	Wing without hyaline spot between *dm*-*cu* and subapical stripe in m_1_ cell, and no hyaline round median spot near CuA_1_ in cua_1_ cell	***Ph* . *uncinata* (Shi et al., 2009)**
5	Antenna scape and pedicel yellow; surstylus with a small digitiform subapical process and a curved claviform inner process; phallus with a pair of subapical lateral processes and a hooked apical process in ventral view	***Ph* . *digitiformis* (Shi et al., 2009)**
–	Antenna scape and pedicel black; surstylus with a broad apical process, a small bulb-like subapical process and a narrow long curved inner process; phallus with a pair of median teeth and a pair of acuate triangular apical processes in ventral view	***Ph* . *lunifera* (de Meijere, 1910)**
6	Frons distinctly longitudinally sunken in middle, with one velvet black median spot	**genus *Prosopophorella* (de Meijere, 1917) 7**
–	Frons raised, with velvet rectangular spot and pruinescence	**genus *Cestrotus* (Loew, 1862) 9**
7	Palpus blackish brown; all femora brown except yellow apically; wing and male genitalia not as follows	**8**
–	Palpus yellow or brownish yellow; mid and hind femora with one brown ventral band at basal 2/3; wing with brown cloud over *r*-*m* separating from one brown spot over R_1_ in subcostal cell and brown spots surrounding *dm*-*cu*; male genitalia: syntergosternite with short setulae and a pair of ventral processes, epandrium with two pairs of long dorsal setae in ventral view	***Pr* . *yoshiyasui* (Sasakawa, 2001)**
8	Mesonotum with two wide black median stripes, a pair of short black lateral bands behind suture and a narrow grayish white pruinescent band along the rows of dorsocentral setae; tarsi 3–5 pale brown; syntergosternite without ventral process; halter pale yellow	***Pr* . *zhuae* (Shi & Yang, 2009)**
–	Mesonotum with one gray pruinescent band and a pair of gray pruinescent bands along the rows of dorsocentral setae; tarsi 3–5 yellow; syntergosternite with a pair of ventral processes; halter white	***Pr* . *longa* sp. nov.**
9	Face with one brown median longitudinal band	**15**
–	Face without brown median bands	**10**
10	Scutellum yellow or with yellow pruinescence, without brown spots	**11**
–	Scutellum with gray or yellow pruinescence, with brown spots basally	**12**
11	Mesonotum with black trapeziform spot posteriorly; wing 2 times longer than wide	***C* . *apicalis* (Hendel, 1920)**
–	Mesonotum with two coterminous yellow trapeziform spots present at posterior 1/3, the trapeziform spots basally with two coterminous round brown spots extending to the base of scutellum; wing 2.5 times longer than wide	***C* . *abdominalis* sp. nov.**
12	Scutellum with two brown spots apically	***C* . *heteropterus* (Shi et al., 2009)**
–	Scutellum without brown spots apically	**13**
13	Face yellow with brown spots	**14**
–	Face white without spots	***C* . *albifacies* sp. nov.**
14	Mesonotum with brown spots on transverse suture; surstylus with outer process twice as long as wide in ventral view	***C* . *flavoscutellat* us (de Meijere, 1910)**
–	Mesonotum with brown spots on transverse suture large and ensiform posteriorly; surstylus with outer process 4 times longer than wide in ventral view	***C* . *longinudus* (Shi et al., 2009)**
15	Palpus yellow; wing distally lacking marginal spots; surstylus with outer process elongate and blunt distally in lateral view	***C* . *liui* (Shi et al., 2009)**
–	Palpus black; wing with distal brown marginal spots between R_2+3_ and R_4+5_ and between R_4+5_ and M_1+2_; surstylus with outer process distinctly triangular in lateral view	**16**
16	Wing with a small rhombic hyaline spot in the brown area between R_2+3_ and R_4+5_; scutellum with paired elongate brown spots confluent with brown patch on mesonotum; surstylus with inner process strongly arched, similar in size to outer process in ventral view	***C* . *acuticurvus* (Shi et al., 2009)**
–	Wing lacking hyaline spot in brown area between R_2+3_ and R_4+5_; scutellum with paired elongate brown spots isolated, separated from brown patch on mesonotum; surstylus with inner process not strongly arched, larger than outer process in ventral view	***C* . *obtusus* (Shi et al., 2009)**

### Species descriptions

#### Cestrotus abdominalis sp. nov.

Taxon classificationAnimaliaDipteraLauxaniidae

4AE18A84-A5F4-5D90-BF3E-8542B3CFABAA

http://zoobank.org/66807266-69FC-49BF-9762-8FD4CE17BFC5

Figures 1–5 , 6–10

##### Type material.

***Holotype***: ♂ (CAUC), China, Yunnan: Menglun, Lvshilin, 5.V.2009, Tingting Zhang.

##### Etymology.

Latin, *abdominalis*, referring to the white abdominal tergites I and II of the new species.

##### Diagnosis.

Face pale yellow, with one tubercle on middle and one rounded tubercle near ventral margin. Frons with one black velvet rectangular spot. Antenna yellow except pedicel blackish brown; arista brown except yellow basally, plumose. Thorax brown with gray pruinescence. Mesonotum with a pair of brown median bands and a pair of undulating lateral bands on anterior margin. Legs yellow, tibia with one incomplete brown ring near base and on tip respectively. Wing r_1_ cell half apically with broad brown band connected with the subapical band of r_2+3_ cell and r_4+5_ cell. Male genitalia: syntergosternite semicircular; epandrium trapeziform in lateral view; surstylus broad basally, tip slender and curved; hypandrium V-shaped.

##### Description.

**Male**. Body length 3.8 mm, wing length 3.9 mm. **Female.** Unknown.

**Figures 1–5.**
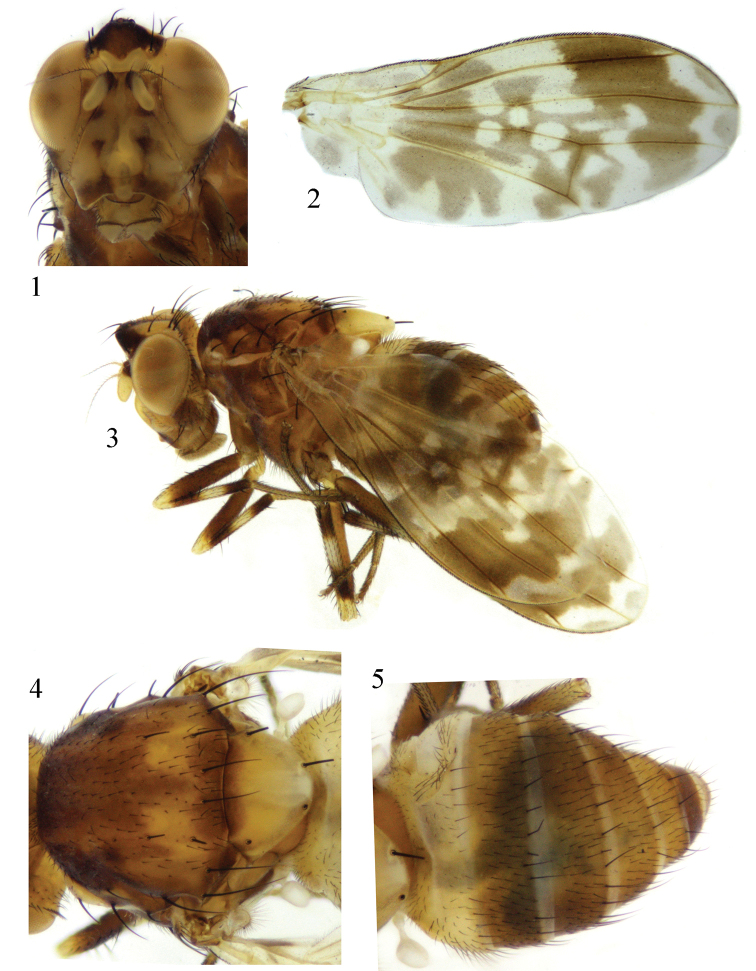
*Cestrotus abdominalis* sp. nov. Male. **1** head, anterior view **2** wing **3** habitus, lateral view **4** thorax, dorsal view **5** abdomen, dorsal view.

Head (Fig. 1) yellow. Face pale yellow, with one tubercle centrally and one rounded tubercle near ventral margin; sides of the central tubercle with one brown spot on dorsal margin and middle respectively, and with a pair of brown lateral longitudinal bands on ventral margin. Frons wider than long and parallel-sided, with one black velvet rectangular spot; ocellar triangle blackish gray, ocellar seta developed, nearly as long as anterior fronto-orbital seta; anterior fronto-orbital seta curved, shorter than posterior fronto-orbital seta. Occiput yellow, with one brown narrow median band extending to ocellar triangle. Parafacial with one triangular brown spot between eye and base of antenna; gena with one brown spot, length of gena and sub-gena about 1/2 eye height. Antenna yellow except pedicel blackish brown; 1^st^ flagellomere about 1.6 times longer than high; arista brown except yellow basally, plumose, the longest ray slightly shorter than 1^st^ flagellomere height. Proboscis yellow except black on margin, with yellow and black setulae; palpus yellow with black setulae.

Thorax (Fig. 4) brown with gray pruinescence. Mesonotum with a pair of brown median bands and a pair of undulating lateral bands on anterior margin, a pair of brown lateral spots present behind scutal suture; two coterminous yellow trapeziform spots present at posterior 1/3, the trapeziform spots basally with two coterminous round brown spots extending to base of scutellum. Three dorsocentral setae; acrostichal setulae in six rows; a pair of prescutellar setae. One anepisternal seta, one katepisternal seta. Scutellum yellow. Legs yellow, femur brown except yellow apically, tibia with one incomplete brown ring near base and on tip respectively. Fore femur with six posterior dorsal setae, four posterior ventral setae, seventeen comb-like anterior ventral setae; tibia with one dorsal preapical seta, one short apical ventral seta. Mid femur with eight anterior setae; tibia with one strong dorsal preapical seta, two strong apical ventral setae. Hind femur with preapical anterior dorsal seta; tibia with one dorsal preapical seta, one short apical ventral seta. Wing (Fig. 2) about 2.5 times longer than wide, hyaline; r_1_ cell half apically with broad brown band connected with the subapical band of r_2+3_ cell and r_4+5_ cell, form wavy band extending to posterior margin; r_2+3_ cell and r_4+5_ cell with pale brown margin spots; hyaline region of *r*-*m* surrounded by “+” shape brown spot; *dm*-*cu* with brown spot, sides of *dm*-*cu* with hyaline spot; costa with 2^nd^, 3^rd^, and 4^th^ sections in proportion of 6.1 : 2.2 : 1; *r*-*m* behind middle of the discal cell; ultimate and penultimate sections of M_1_ in proportion of 2.1 : 1; ultimate section of CuA_1_ about 1/8 of penultimate section. Halter white.

**Figures 6–10.**
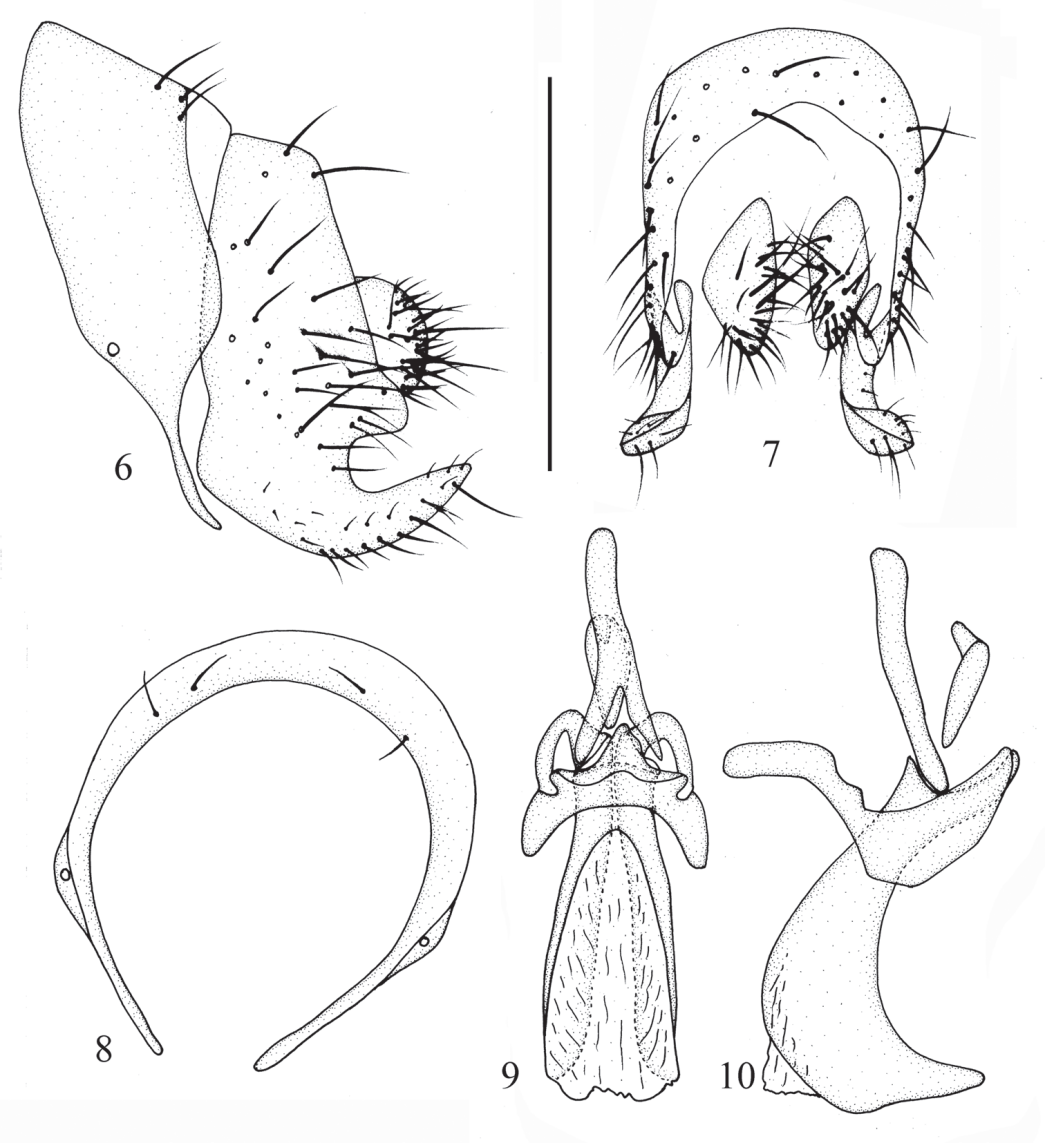
*Cestrotus abdominalis* sp. nov. Male. **6** syntergosternite and epandrium, lateral view **7** epandrial complex, posterior view **8** syntergosternite, anterior view **9** aedeagal complex, ventral view **10** aedeagal complex, lateral view. Scale bar: 0.5 mm.

Abdomen (Fig. 5) with gray pruinescence; tergites I and II white, tergites III-IX brown. Male genitalia (Figs 6–10): syntergosternite semicircular, broad half dorsally and narrow half ventrally. Epandrium trapeziform in lateral view. Surstylus extending from the base of tergite, broad basally, tip slender and curved, surstylus curved outwards in posterior view. Hypandrium V-shaped. Gonopod vestigial. Phallus without apical concave, with a pair of dorsal sclerites, tip of the sclerites slender and curved in lateral view, broad and deep. Phallapodeme shorter than phallus.

##### Remarks.

The new species is similar to *Cestrotus acuticurvus* Shi, Yang & Gaimari, 2009 from China (Yunnan) in having spots on the face and wing, but the latter has a mesonotum with brown trapeziform spots and a dm-*cu* with a brown spot.

##### Distribution.

China (Yunnan).

#### Cestrotus albifacies sp. nov.

Taxon classificationAnimaliaDipteraLauxaniidae

E37EF95E-65CB-50D8-AA6A-772BB636F9B5

http://zoobank.org/0501BC02-D746-48BF-BAE7-7FA60F31BF3E

Figures 11–15 , 16–20

##### Type material.

***Holotype***: ♂ (CAUC), China, Yunnan: Hekou, Nanxi Town, 132 m, 22.V.2009, Guoquan Wang.

##### Etymology.

Latin, *albifacies*, referring to the new species’ white face without any spots or bands.

##### Diagnosis.

Face white, without brown spot or band. Frons with a pair of black velvet triangular spots. Antenna yellow; arista blackish brown except brown basally, plumose. Thorax with yellow pruinescence. Mesonotum with a pair of elliptical brown spots present on scutal suture. Legs yellow, femur brown except yellow apically; tibia with one incomplete brown ring near base. Wing r_2+3_ cell and r_4+5_ cell without margin spot; the hyaline region of *dm*-*cu* surrounded by two brown spots. Male genitalia: syntergosternite circular; surstylus consisting of one outer process and inner process; hypandrium Y-shaped, inner process longer than phallapodeme.

##### Description.

**Male.** Body length 3.3 mm, wing length 3.0 mm. **Female.** Unknown.

**Figures 11–15.**
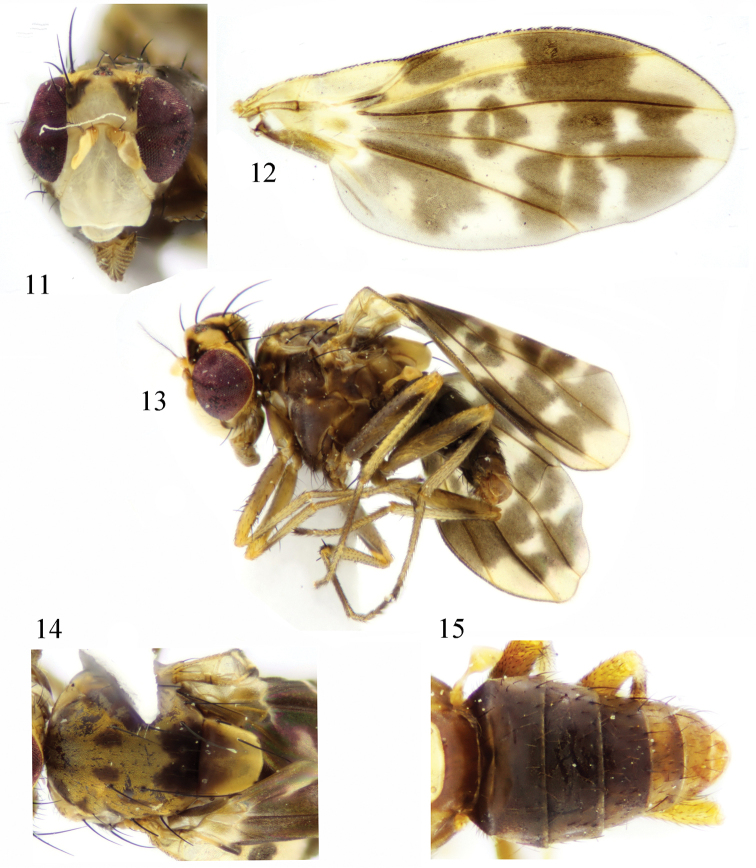
*Cestrotus albifacies* sp. nov. Male. **11** head, anterior view **12** wing **13** habitus, lateral view **14** thorax, dorsal view **15** abdomen, dorsal view.

Head (Fig. 11) yellow. Face white, without brown spot or band. Frons wider than long and parallel-sided, with a pair of black velvet triangular spots; ocellar triangle blackish gray; ocellar setae broken, anterior fronto-orbital seta curved, shorter than posterior fronto-orbital seta. Occiput yellow, with one blackish brown median band extending to ocellar triangle. Gena yellow, with one kidney-shape brown spot; length of gena and sub-gena about 1/2 eye height. Antenna yellow; 1^st^ flagellomere about 1.6 times longer than high; arista blackish brown except brown basally, plumose, the longest ray slightly longer than 1^st^ flagellomere width. Proboscis brown, with yellow and black setulae; palpus yellow with black setulae.

Thorax (Fig. 14) blackish brown with yellow pruinescence. Mesonotum with a pair of brown median bands and a pair of undulating lateral bands on anterior margin, a pair of elliptic brown spots present on scutal suture; one black trapeziform spot present at posterior 1/3, anterior margin of the spot bifurcated. Three dorsocentral setae; acrostichal setulae in six rows; a pair of prescutellar setae, shorter than the first dorsocentral seta. One anepisternal seta, one katepisternal seta. Scutellum yellow with gray pruinescence, one brown trapeziform spot present on half basally and connect with the spot of mesonotum, posterior margin of the spot bifurcated. Legs yellow, femur brown except yellow apically; tibia with one incomplete brown ring near base; the fifth tarsus brown. Fore femur with six posterior dorsal setae, four posterior ventral setae, twelve comb-like anterior ventral setae; tibia with one dorsal preapical seta, one short apical ventral seta. Mid femur with eight anterior setae; tibia with one strong dorsal preapical seta, two strong apical ventral setae. Hind femur with preapical anterior dorsal seta; longer than wide, hyaline; r_1_ cell half apically with broad brown band connected with the subapical band of r_2+3_ cell and r_4+5_ cell, form wavy band extending to posterior margin; r_2+3_ cell and r_4+5_ cell without margin spot; the hyaline region of *r*-*m* surrounded by “+” shape brown spot; the hyaline region of *dm*-*cu* surrounded by two brown spots; costa with 2^nd^, 3^rd^ and 4^th^ sections in proportion of 4.3 : 1.9 : 1; *r*-*m* behind middle of the discal cell; ultimate and penultimate sections of M_1_ in proportion of 2.0 : 1; ultimate section of CuA_1_ about 1/7 of penultimate section. Halter yellow.

**Figures 16–20.**
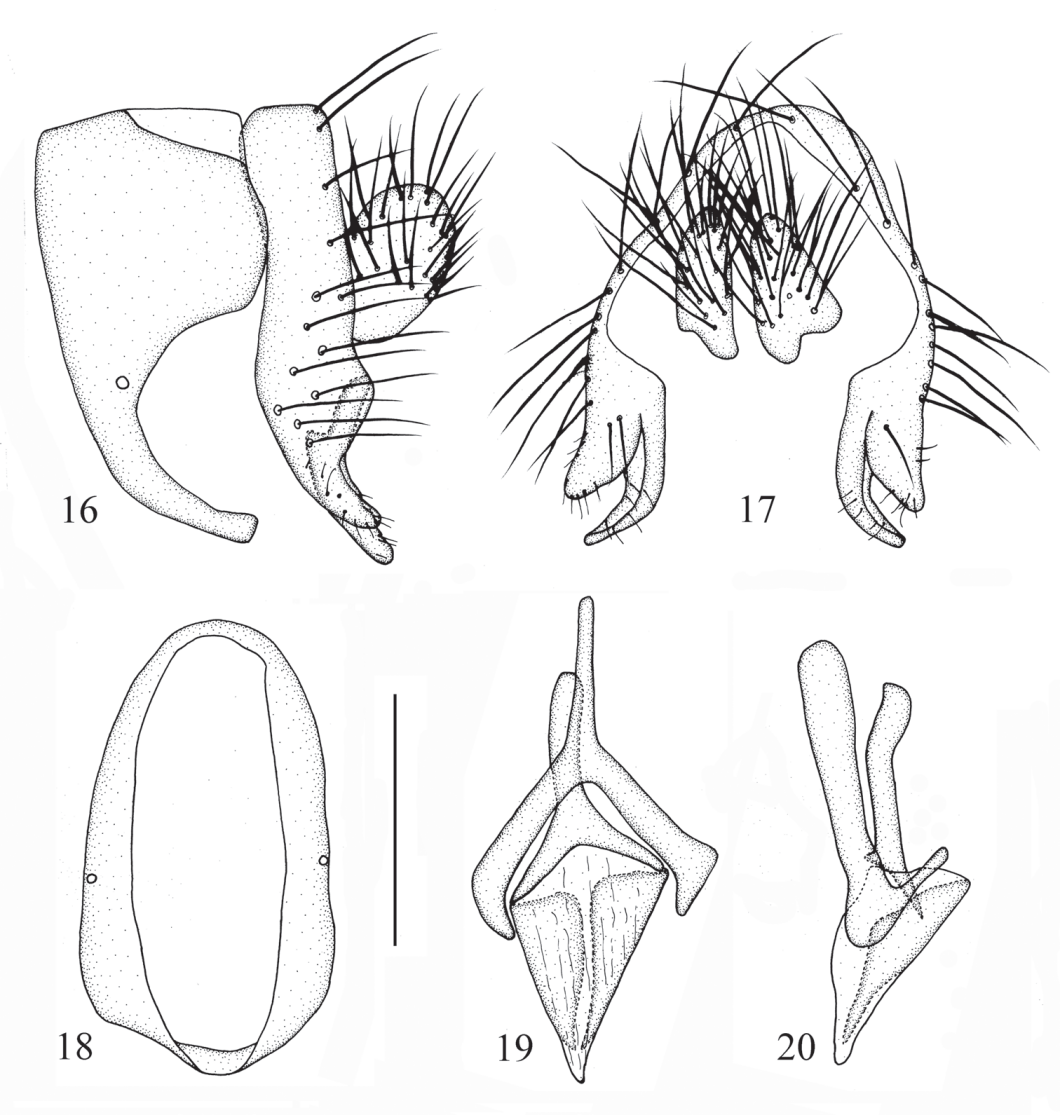
*Cestrotus albifacies* sp. nov. Male. **16** syntergosternite and epandrium, lateral view **17** epandrial complex, posterior view **18** syntergosternite, anterior view **19** aedeagal complex, ventral view **20** aedeagal complex, lateral view. Scale bar: 0.2 mm.

Abdomen (Fig. 15) with gray pruinescence; tergites I–IV blackish brown, tergites V–IX brownish yellow. Male genitalia (Figs 16–20): syntergosternite circular, broad half dorsally and narrow half ventrally. Epandrium long and narrow in lateral view. Surstylus consisting of one outer process and inner process, the processes similar in shape, rod-like and with setulae, curved in lateral view, inner process longer than outer process in posterior view. Hypandrium Y-shaped, inner process longer than phallapodeme. Gonopod vestigial. Phallus acute apically, without apical concave, with a pair of triangular dorsal sclerites. Phallapodeme as long as phallus.

##### Remarks.

The new species is similar to *Cestrotus heteropterus* Shi, Yang & Gaimari, 2009 from China (Yunnan) in the color of the face and in having spots on the tergite and wing, but the latter has spots on the face and the antennal pedicel brown.

##### Distribution.

China (Yunnan).

#### Phobeticomyia motuoensis sp. nov.

Taxon classificationAnimaliaDipteraLauxaniidae

B370DC94-859C-5180-B6DA-B5260E807917

http://zoobank.org/13B8BB91-A697-4F68-BA24-207B7A2008F4

Figures 21–25 , 26–30

##### Type material.

***Holotype***: ♂ (CAUC), China, Tibet: Motuo County, 1100 m, 26.VII.2012, Wenliang Li. ***Paratypes***: 1♂ (CAUC), China, Tibet: Motuo County, 1100 m, 28.VII.2012, Wenliang Li; 2♀♀ (CAUC), China, Tibet: Motuo County, 1100 m, 28.VII.2012, Xuankun Li.

##### Etymology.

Latinized, referring to the type locality of the new species.

##### Diagnosis.

Face shining, blackish brown on half basally, yellow on half apically, with a pair of blackish brown trapeziform spots. Frons with two blackish brown velvet longitudinal bands extending to sides of ocellar triangle. Thorax brown with gray pruinescence. Mesonotum with two brown median bands, one gray narrow band between the median bands. Legs blackish brown, hind tibia pale brown, all tibiae with one white ring near tip. Wing brown, with three hyaline spots between R_2+3_ and R_4+5_. Male genitalia: syntergosternite semicircular; hypandrium V-shaped, with a pair of short inner process on sides, one V-shaped membrane on inferior margin; gonopod with setulae basally, curved apically.

##### Description.

**Male.** Body length 3.7–3.8 mm, wing length 3.7–3.8 mm. **Female.** Body length 3.8–4.0 mm, wing length 3.9–4.1 mm.

**Figures 21–25.**
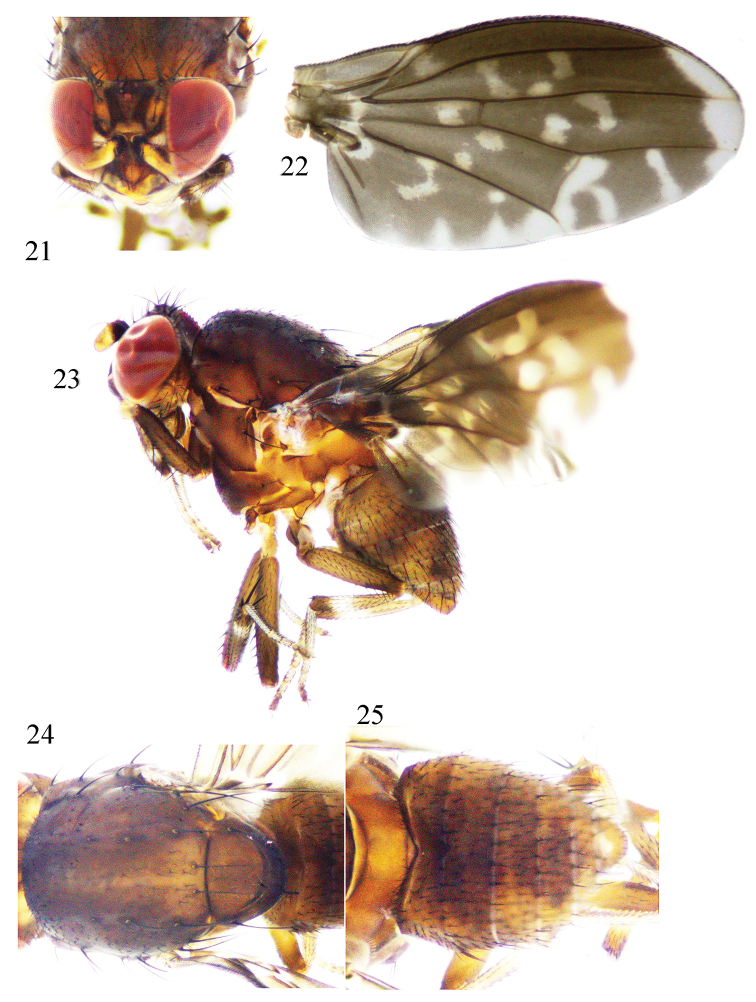
*Phobeticomyia motuoensis* sp. nov. Male. **21** head, anterior view **22** wing **23** habitus, lateral view **24** thorax, dorsal view **25** abdomen, dorsal view.

Head (Fig. 21) brownish yellow. Face shining, blackish brown on half basally, yellow on half apically, with a pair of blackish brown trapeziform spots; parafacial black on half basally, yellow on half apically, with silvery pruinescence. Frons yellow, wider than long and parallel-sided, with two blackish brown velvet longitudinal bands extending to sides of ocellar triangle, the bands connected with one W-shape transverse band; ocellar triangle brown, ocellar seta developed, longer than anterior fronto-orbital seta; anterior fronto-orbital seta curved, shorter than posterior fronto-orbital seta. Gena about 1/3 eye height. Antenna yellow except pedicel and scape black; 1^st^ flagellomere about 1.7 times longer than high; arista dark brown except pale brown basally, plumose, the longest ray as long as 1^st^ flagellomere height. A silvery spot present between eye and base of antenna. Proboscis brown, with yellow and black setulae; palpus black with black setulae.

Thorax (Fig. 24) brown with gray pruinescence. Mesoscutum with two brown median bands, one gray narrow band between the median bands; two brown lateral bands behind scutal suture, along rows of dorsocentral setae with one gray narrow band respectively. Three dorsocentral setae, with brown basal spots; acrostichal setulae in eight rows, pubescent; a pair of prescutellar setae, as long as the first dorsocentral seta. One anepisternal seta, two katepisternal seta. Scutellum with brownish yellow pruinescence. Legs blackish brown, hind tibia pale brown, all tibiae with one white ring near tip; fore and hind tibia each with one unobvious white basal ring; tarsi pale yellow, the fifth tarsus brown. Fore femur with eight posterior dorsal setae, five posterior ventral setae, fourteen comb-like anterior ventral setae; tibia with one dorsal preapical seta, one short apical ventral seta. Mid femur with five anterior setae; tibia with one strong dorsal preapical seta, three apical ventral setae. Hind femur with one weak preapical dorsal seta, one row anterior ventral setae; tibia with one weak dorsal preapical seta, one short apical ventral seta. Wing (Fig. 22) brown, with one hyaline apical band on outer margin, three hyaline spots present between R_2+3_ and R_4+5_; *r*-*m* without hyaline spot, the cell in front of *r*-*m* with hyaline spot; discal medial cell with two round hyaline spots; *dm*-*cu* with one hyaline spot outside, and connected with hyaline apical spot of CuA_1_ and hyaline subapical band of m_1_ cell; m_1_ cell with two hyaline subapical bands, one undulating hyaline along the posterior margin of cua_1_; subcostal cell with hyaline spot; costa with 2^nd^, 3^rd^ and 4^th^ sections in proportion of 4.6 : 1.7 : 1; *r*-*m* behind middle of the discal cell; ultimate and penultimate sections of M_1_ in proportion of 1.4 : 1; ultimate section of CuA_1_ about 1/7 of penultimate section. Halter white.

**Figures 26–30.**
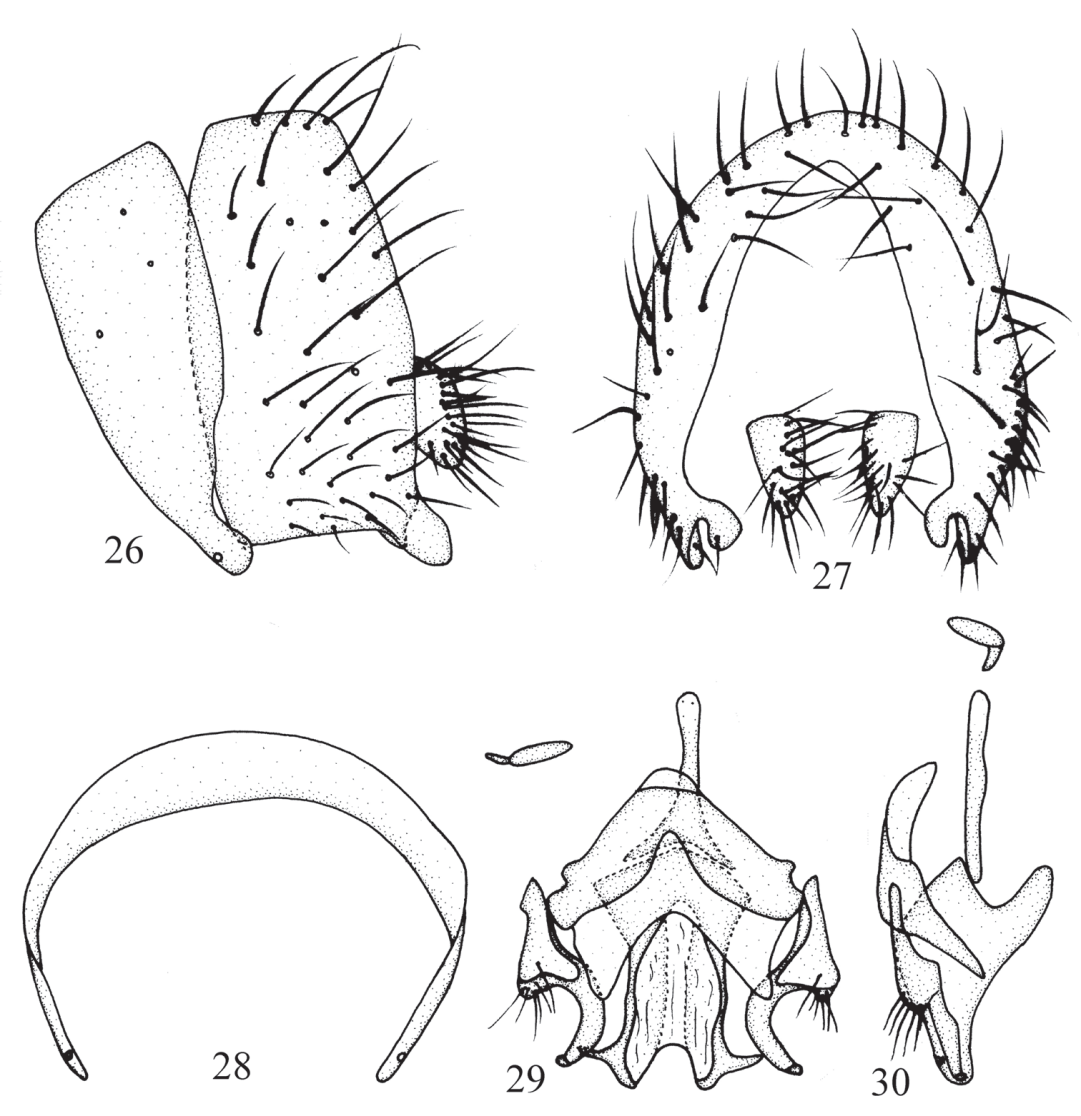
*Phobeticomyia motuoensis* sp. nov. Male. **26** syntergosternite and epandrium, lateral view **27** epandrial complex, posterior view **28** syntergosternite, anterior view **29** aedeagal complex, ventral view **30** aedeagal complex, lateral view.

Abdomen (Fig. 25) brown with gray pruinescence. Male genitalia (Figs 26–30): syntergosternite semicircular. Epandrium near rectangle in lateral view. Surstylus consisting of one short broad outer apical process and one short inner process, the inner process blunt apically and curved in lateral view. Hypandrium V-shaped, with a pair of short inner process on sides, one V-shape membrane on inferior margin. Gonopod with setulae basally, curved apically. Phallus with apical concave and a pair dorsal sclerites, a pair of near apical process present on sides. Phallapodeme shorter than phallus.

##### Remarks.

The new species is similar to *Phobeticomyia lunifera* (de Meijere, 1910) from Indonesia (Java), but the latter has no inner process on the hypandrium, and the phallus has no apical spine.

##### Distribution.

China (Tibet).

#### Prosopophorella longa sp. nov.

Taxon classificationAnimaliaDipteraLauxaniidae

0A5EE479-A7DE-5EC1-9D15-E1B2842E29B4

http://zoobank.org/A0CAF592-59C9-4590-B188-B36C50DA6702

Figures 31–36 , 37–41

##### Type material.

***Holotype***: ♂ (CAUC), China, Tibet: Motuo County, 1100 m, 26.VII.2012, Xuankun Li. ***Paratypes***: (CAUC). 2♀♀ (CAUC), data same as above; 2♀♀ (CAUC), China, Tibet: Motuo County, 1100 m, 26.VII.2012, Wenliang Li.

##### Etymology.

Latin, *longa*, meaning long, referring to the new species’ clypeus with one long median process.

##### Diagnosis.

Face brownish yellow, with one brown broad median band on middle. Frons with one yellow W-shape spot on anterior margin. Antenna blackish brown; arista brown, pubescent. With one long median process on male clypeus. Thorax with gray pruinescence. Mesonotum with one narrow gray pruinescent band on the middle and a pair of gray pruinescent bands along the rows of dorsocentral setae. Legs yellow; tibiae with blackish brown sub-basal ring and apical ring. Wing with one irregular hyaline spot under the tip of R_2+3_, one hyaline apical spot between R_4+5_ and M_1_. Male genitalia: syntergosternite broad, circular, with a pair of short ventral process; surstylus short, rod-like in lateral view; aedeagal dorsal sclerites consisting of a pair of sclerites, narrow apically.

##### Description.

**Male.** Body length 6.5 mm, wing length 6.4 mm. **Female.** Body length 6.5–6.7 mm, wing length 6.4–6.5 mm.

Head (Figs 34, 35) yellow. Face brownish yellow, with one brown broad median band on middle, a pair of triangular blackish brown lateral spots on ventral-lateral angle; parafacial black on inner margin. Frons yellow, wider than long and parallel-sided, with one yellow W-shape spot on anterior margin, two triangular black spots on half apically; the middle with one black velvet rectangular spot extending to vertex, and connected with the triangular black spot on lateral margin; ocellar triangle black, ocellar seta developed, longer than anterior fronto-orbital seta; anterior fronto-orbital seta curved. Occiput yellow, male with one broad brown median band connected with black velvet rectangular spot. Gena yellow, with one brown rectangular spot; gena about 1/3 eye height. Antenna blackish brown, 1^st^ flagellomere yellow except brown on dorsal margin and tip; 1^st^ flagellomere about 1.2 times longer than high; arista brown, pubescent. The black band of inner margin between eye and antenna nearly triangular, connected with the black triangular spot of frons lateral margin. Clypeus black, with one long median process. Proboscis yellow except blackish brown apically, with yellow and black setulae; palpus blackish brown with black setulae.

Thorax (Fig. 36) blackish brown with gray pruinescence. Mesonotum with one narrow gray pruinescent band on the middle and a pair of gray pruinescent bands along the rows of dorsocentral setae. Three dorsocentral setae, the most anterior dorsocentral setae near scutal suture; acrostichal setulae in six rows, pubescent; a pair of prescutellar setae, shorter than the first dorsocentral seta. One anepisternal seta, one katepisternal seta. Legs yellow; coxa pale brown; femora brown except yellow apically; tibiae with blackish brown sub-basal ring and apical ring. Fore femur with five posterior dorsal setae, two posterior ventral setae, 27 comb-like anterior ventral setae; tibia with one weak dorsal preapical seta, one short apical ventral seta. Mid femur with eight anterior setae; tibia with one strong dorsal preapical seta, two apical ventral setae. Hind tibia with one dorsal preapical seta, one short apical ventral seta. Wing (Fig. 33) about 3.1–3.3 times longer than wide, hyaline, with one narrow brown sub-basal band connect with gray posterior margin, one broad brown median band extending to *dm*-*cu*, one brown subapical band connect with gray posterior margin, one hyaline band between brown subapical band and median band; one irregular hyaline spot present under the tip of R_2+3_, one hyaline apical spot between R_4+5_ and M_1_; *r*-*m* and *dm*-*cu* with narrow hyaline bands, surround by brown cloud-like spot; subcostal cell with brown spot apically, the spot through R_1_; costa with 2^nd^, 3^rd^ and 4^th^ sections in proportion of 8.2 : 3.1 : 1; *r*-*m* behind middle of the discal cell; ultimate and penultimate sections of M_1_ in proportion of 2.2 : 1; ultimate section of CuA_1_ about 1/8 of penultimate section. Halter white.

Abdomen (Fig. 36) blackish brown. Male genitalia (Figs 37–41): syntergosternite broad, circular, with a pair of short ventral process. Epandrium near rectangle in lateral view. Surstylus short, rod-like in lateral view. Hypandrium V-shaped, with a pair of inner processes and a pair of lateral processes. Gonopod short. Aedeagal dorsal sclerites consisting of a pair of sclerites, narrow apically; apical concave deep.

**Figures 31–36.**
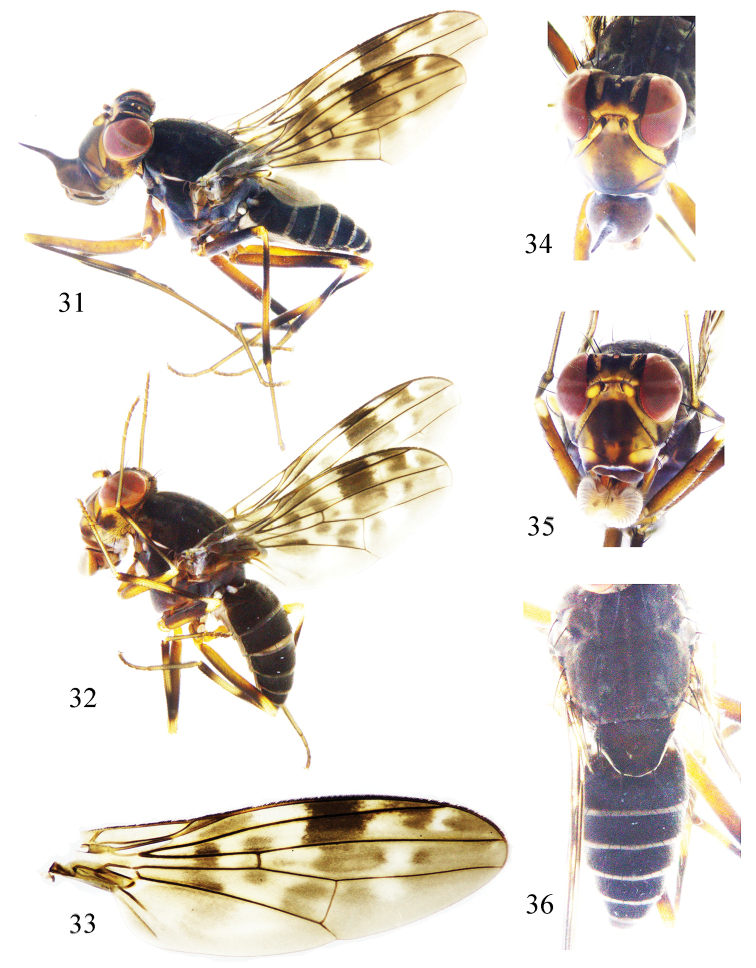
*Prosopophorella longa* sp. nov. Male. **31** habitus, lateral view **33** wing **34** head, anterior view **36** thorax and abdomen, dorsal view; female. **32** habitus, lateral view **35** head, anterior view.

##### Remarks.

The new species is similar to *Prosopophorella zhuae* Shi & Yang, 2009 from China (Guangxi) in body color, face, wing type and male genitalia. However, tarsi 3–5 of the latter is pale brown and the syntergosternite has no ventral process, whereas tarsi 3–5 in the new species is yellow and the syntergosternite has a pair of ventral processes.

**Figures 37–41.**
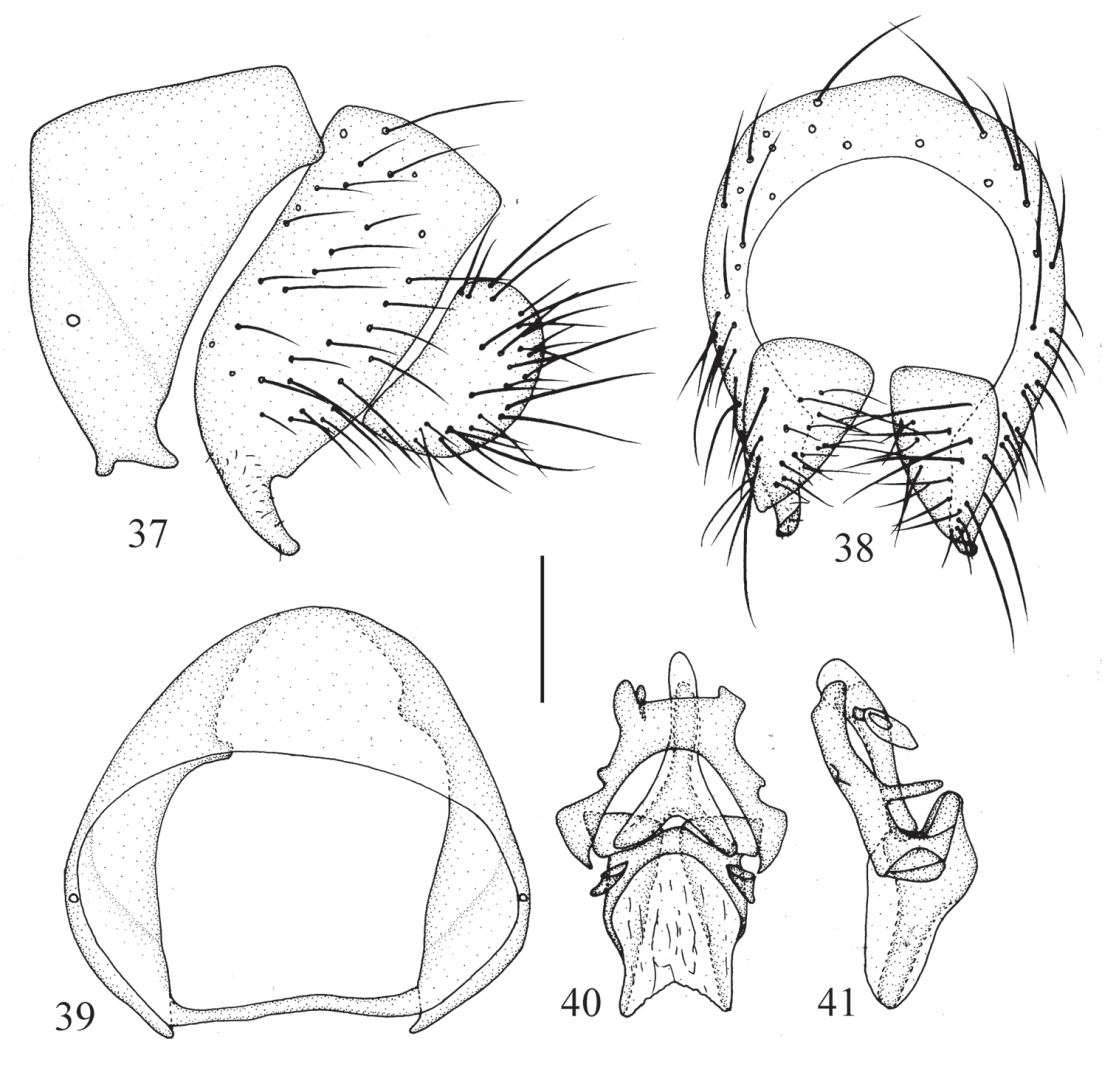
*Prosopophorella longa* sp. nov. Male. **37** syntergosternite and epandrium, lateral view **38** epandrial complex, posterior view **39** syntergosternite, anterior view **40** aedeagal complex, ventral view **41** aedeagal complex, lateral view. Scale bar: 0.2 mm.

##### Distribution.

China (Tibet).

## Supplementary Material

XML Treatment for Cestrotus abdominalis

XML Treatment for Cestrotus albifacies

XML Treatment for Phobeticomyia motuoensis

XML Treatment for Prosopophorella longa

## References

[B1] de Meijere JCH (1910) Studien über südostasiatische Dipteren IV. Die neue Dipterenfauna van Krakatau. Tijdschrift voor Entomologie 53: 58–194. 10.5962/bhl.title.8578

[B2] Evenhuis NL (1989) Catalog of the Diptera of the Australasian and Oceanian Regions. American Entomologist 38(3): 182–183. 10.1093/ae/38.3.182

[B3] Gaimari SD Silva VC (2010) Lauxaniidae (Lauxaniid flies). In: Brown BV, Borkent A, Cumming JM, Wood DM, Woodley NE (Coords) Manual of Central American Diptera. Vol. 2. NRC Research Press, Ottawa, Ontario, Canada, 971–995.

[B4] Hendel F (1920) Neue *Cestrotus*-Arten des ungarischen Nationalmuseums (Dipt., Lauxaniid). Verhandlungen der Zoologisch-Botanischen Gesellschaft in Wien 20: 74–80.

[B5] Kertész K (1915) H. Sauters Formosa-Ausbeute. Lauxaniidae. II. Annales Musei Nationalis Hungarici 13: 491–534.

[B6] Li WL Qi L Yang D (2020) Three new species of the genus *Noonamyia* Stuckenberg 1971 (Diptera: Lauxaniidae) from China. Oriental Insects. 10.1080/00305316.2020.1754955

[B7] Loew H (1862) Bidrag till kännedomen om Afrikas Diptera. Öfversigt af Kongliga Vetenskaps-Akademiens Förhandlingar 19: 3–14.

[B8] Sasakawa M (1987) Lauxaniidae of Thailand (Diptera) part 1. Akitu, new series 92: 1–9.

[B9] Sasakawa M (2001) Oriental Lauxaniidae (Diptera) (Part 2): Fauna of the Lauxaniidae of Vienam. Scientific Reports of Kyoto Prefectural University, Human Environment and Agriculture 53: 39–94.

[B10] Shi L Yang D (2009a) Two new species of the genus *Noonamyia* from Hainan in China (Diptera, Lauxaniidae). Zootaxa 2014: 34–40. 10.11646/zootaxa.2014.1.3

[B11] Shi L Yang D (2009b) Species of the genus *Prosopophorella* from China (Diptera: Lauxaniidae). Annals Zoologici 59(2): 159–164. 10.3161/000345409X463967

[B12] Shi L Li WL Yang D (2009a) Five new species of the genus *Dioides* from China (Diptera, Lauxaniidae). Annals Zoologici 59(1): 93–105. 10.3161/000345409X432600

[B13] Shi L Li WL Yang D (2009b) Two new species of the genus *Phobeticomyia* from China (Diptera, Lauxaniidae). Zootaxa 2090: 57–68. 10.11646/zootaxa.2090.1.3

[B14] Shi L Yang D Gaimari SD (2009c) Species of the genus *Cestrotus* Loew from China (Diptera: Lauxaniidae). Zootaxa 2009: 41–68. 10.11646/zootaxa.2009.1.4 28610070

[B15] Shi L Yang D (2014) Supplements to species groups of the subgenus Homoneura in China (Diptera: Lauxaniidae: *Homoneura*), with descriptions of twenty new species. Zootaxa 3890(1): 1–117. 10.11646/zootaxa.3890.1.1 25544372

[B16] Stuckenberg BR (1971a) A review of the Old World genera of Lauxaniidae (Diptera). Annals of the Natal Museum 20(3): 499–610.

[B17] Stuckenberg BR (1971b) An account of the genus Noeetomima with descriptions of new species from Queensland and Nepal (Diptera: Lauxaniidae). Annals of the Natal Museum 21(1): 21–28.

[B18] Van der Wulp (1891) Eenige Uitlandsche Diptera. Tijdschrift voor Entomologie 34: 193–215.

